# Should Hospital Patients Have Bedside Access to Their Complete Medical Records?

**DOI:** 10.2196/jopm.8844

**Published:** 2017-05-17

**Authors:** Lisa V Grossman, Susan Restaino, David Vawdrey

**Keywords:** Acute patient portals, patient portals, PHR, medical records, transparency, note-sharing

Steven Jones finally received his heart transplant just two months shy of his 51st birthday. After 7 months spent lying in a hospital bed, unable to walk, transplant felt like the light at the end of the tunnel. “I thought life would return to normal after transplant,” said Mr. Jones. “But that just wasn’t true.”

The Heart Institute at Columbia University Medical Center provides care for cardiology patients, including heart transplant recipients. In August 2016, the institute began a first-of-its-kind initiative. Patients received bedside access to their entire medical record, including clinical notes, through an online patient portal.

After Mr. Jones’s transplant, a series of rejection events complicated his clinical course. While hospitalized at Columbia University Medical Center, Mr. Jones volunteered for the initiative. “I thought I knew everything about my disease,” he recalled. “But when my doctor offered to let me read my notes, I learned just how much I didn’t know.”

Two years ago, the federal electronic health record financial incentive program “meaningful use” prompted rapid adoption of online patient portals. Per meaningful use, hospitals must permit patients “to view online, download, and transmit their health information.” In August 2014, just 10.4% of US hospitals met this requirement. By November 2015, 64.3% did. Because meaningful use requires that health information be released “within 36 hours of discharge,” hospitals generally do not permit or encourage inpatient access.

Yet, clinicians and patients increasingly view full transparency as a moral imperative. Patient advocacy to access medical records and even participate in note writing began in the 1970s, concurrent with the medical movement rejecting paternalism. The 1996 Health Insurance Portability and Accountability Act guarantees patients’ right to review their medical data. Proponents of transparency believe online patient portals actualize the HIPAA mandate, by overcoming barriers such as time delays and photocopying costs.

The OpenNotes consortium reports that over 10 million individuals now have electronic access to their primary care providers’ office notes. In OpenNotes trials, four out of five subjects accessed their physicians’ notes online when given the opportunity to do so [1]. In spite of OpenNotes’ success, electronic note-sharing remains relatively unstudied outside primary care settings.

## The Note-Sharing Initiative at Columbia University Medical Center

In a randomized controlled trial [2], our team introduced a bedside portal to cardiology inpatients at Columbia University Medical Center. The bedside portal incorporates multiple features, including medication summaries, diagnostic test results, and the inpatient care team.

The note-sharing initiative at the Heart Institute provided 10 patients with real-time access to their complete medical record on tablet computers. We utilized a modified version of our bedside portal that included physician notes. We reviewed system usage logs and performed qualitative interviews to evaluate patients’ experiences.

Some participants reported initial anxiety about viewing their medical record. One patient reluctantly agreed to participate, and initially said: “I don’t think I will look [at the portal], because I’d rather not know.” But the next day, he said: “I felt anxious at first, but now I’m starting to look. I like to watch my weight go down—it makes me feel good to see how much fluid I’m losing…I don’t understand everything in the notes, but it’s amazing to see everything that goes into my care.”

Most participants reported enthusiasm about viewing their record. Half voluntarily requested access outside the hospital, and one participant even volunteered to pay for access. He said: “I already learned how to use this [portal] here [in the hospital]…I don’t want to use new software at home.”

Participants navigated to the “clinical notes” feature most frequently, and spent more time using this feature than any other. One participant observed: “The notes were where I was really able to find out what was going on, where all the information was put together…I love being up to speed with [my physician]. When she comes in, she doesn’t have to explain what’s going on, because I already know.”

Participants reported that portal access impacted their care. Mr. Jones related an incident where he noticed that prednisone had fallen off of his medication list. “I showed the nurse,” he said, “who agreed with me that something wasn’t right. She called the doctor, and within a minute and a half the prednisone was back on [my medication list]. And within another minute and a half, my nurse was back with the [prednisone] pill.”

## The Future of Transparency for the Medical Community

Patients benefit from having access to their complete medical record, including physician notes. Information can empower patients to participate in their care, and raise their awareness of providers’ actions performed on their behalf. Information also lessens the anxiety, disempowerment, and suffering patients experience due to uncertainty about their condition. Our participants demonstrated a willingness to engage with complex information. This finding is consistent with previous research demonstrating that usage rates for note-sharing patient portals exceed rates for simpler portals [3,4].

Both proponents and opponents of medical record transparency support their arguments with strong ethical principles. Opponents argue that medical record information is too complex or too alarming for patients, and that full transparency violates the “first, do no harm” principle. Proponents reject such rhetoric as paternalistic, and support full transparency under the autonomy principle. Dr. Donald Berwick recently wrote that “anything professionals know about their work, the people and communities they serve can know, too, without delay, cost, or smokescreens” [5].

The pernicious effect of computers on the doctor-patient relationship is a widely cited problem in modern medicine. Transparency reinvents the computer as a tool to enhance, not detract from, the doctor-patient relationship [6]. Previous research suggests that transparency especially promotes greater trust among vulnerable patient populations [7]. Referring to the broader health care system, Mr. Jones said: “I don’t trust [it], so I’m happy about this information [on the portal]…I feel better able to cope.”

As value-based payment programs gain momentum, an era of consumer-driven health care may be imminent. The question, then, becomes not “if” hospitals will provide real-time access to patients’ complete medical records, but rather “how” and “when.” We owe it to our patients and to ourselves to thoughtfully research transparency and its associated ethical concerns. All of us strive to give patients the best possible information, and if we discover that transparency furthers this goal, we must provide it.

## Authors’ Note

Patients’ names in this paper were changed to preserve anonymity. Quotations were edited for clarity.
